# Exploring the Biological Mechanism of Huang Yam in Treating Tumors and Preventing Antitumor Drug-Induced Cardiotoxicity Using Network Pharmacology and Molecular Docking Technology

**DOI:** 10.1155/2021/9988650

**Published:** 2021-08-25

**Authors:** Hui Zhang, Wenchao Dan, Qingyong He, Jianbo Guo, Shuang Dai, Xiaoshan Hui, Peipei Meng, Qianqian Cao, Wingyan Yun, Xinyuan Guo

**Affiliations:** ^1^Department of Cardiology, Guang'an Men Hospital, China Academy of Chinese Medical Sciences, No. 5, North Line Pavilion, Xicheng District, Beijing 100053, China; ^2^Graduate School of Beijing University of Chinese Medicine, Beijing 100029, China; ^3^The University of Hong Kong, Hong Kong 999077, China; ^4^Department of Radiation Therapy, Cancer Hospital Chinese Academy of Medical Sciences, No. 17, South Panjiayuan, Chaoyang District, Beijing 100021, China

## Abstract

Drugs for the treatment of tumors could result in cardiotoxicity and cardiovascular diseases. We aimed to explore the anticancer properties of Huang yam as well as its cardioprotective properties using network pharmacology and molecular docking technology. The cardiovascular targets of the major chemical components of Huang yam were obtained from the following databases: TCMSP, ETCM, and BATMAN-TCM. The active ingredients of Huang yam were obtained from SwissADME. The cardiovascular targets of antitumor drugs were obtained using GeneCards, OMIM, DrugBank, DisGeNET, and SwissTargetPrediction databases. The drug-disease intersection genes were used to construct a drug-compound-target network using Cytoscape 3.7.1. A protein-protein interaction network was constructed using Cytoscape's BisoGenet, and the core targets of Huang yam were screened to determine their antitumor properties and identify the cardiovascular targets based on topological parameters. Potential targets were imported into the Metascape platform for GO and KEGG analysis. The results were saved and visualized using R software. The components with higher median values in the network were molecularly docked with the core targets. The network contained 10 compounds, including daucosterol, delusive, dioxin, panthogenin-B, and 124 targets, such as TP53, RPS27A, and UBC. The GO function enrichment analysis showed that there were 478 items in total. KEGG enrichment analysis showed a total of 140 main pathways associated with abnormal transcription of cancer, PI3K-Akt signaling pathway, cell cycle, cancer pathway, ubiquitination-mediated proteolysis, and other pathways. Molecular docking results showed that daucosterol, delusive, dioxin, and panthogenin-B had the highest affinity for TP53, RPS27A, and UBC. The treatment of diseases using traditional Chinese medicine encompasses multiple active ingredients, targets, and pathways. Huang yam has the potential to treat cardiotoxicity caused by antitumor drugs.

## 1. Introduction

Drugs for the treatment of tumors could result in cardiotoxicity and cardiovascular diseases, among other side effects [1]. The rapid development of antitumor treatments and the popularization of precision treatments have led to the prolongation of the survival time of patients with cancer. However, their risk of developing cardiovascular disease is increasing because its incidence is significantly correlated with age, and radiotherapy and chemotherapy can induce its development [2]. In 2016, the European Society of Cardiology Tumor Treatment and Cardiovascular Toxicity Collaborative Group compiled the first guideline, the 2016 ESC Tumor Treatment and Cardiovascular Toxicity Statement [3], which clearly indicated that antitumor chemotherapy can lead to coronary atherosclerosis. Moreover, antitumor radiotherapy has a 10% chance of causing heart valve disease, whereas various antitumor chemical drugs can cause arrhythmias.

At present, cardiovascular disease is one of the main factors that affect tumor survival [2]. Interestingly, most of the drugs used to treat tumors are cardiotoxic and can cause cardiovascular diseases. The drugs that cause cardiovascular damage are mainly anthracyclines and their derivatives, such as doxorubicin, idarubicin, epirubicin, and mitoxantrone. Anticancer drugs such as antimetabolites, alkylating agents, and proteasome inhibitors can also cause heart failure [4]. Anthracyclines such as doxorubicin can inhibit mitochondrial activity and sarcoplasmic reticulum enzyme activity. It produces myocardial toxicity in myocardial cells, causing necrosis or apoptosis of myocardial cells, causing cardiac insufficiency or heart failure [3, 5]. Therefore, it is imperative that drugs are developed to simultaneously treat tumors and cardiovascular diseases.

Huang yam is a traditional Chinese medicine and a major ingredient of Di'aoxinxuekang, which is a Chinese patented medicine approved by the European Union [6]. Pharmacological studies [7] have shown that the chemical components of Huang yam are mainly steroidal saponins, such as diosgenin, trillium saponins, slender saponins, *β*-sitosterol, and daucosterol. Huang yam is the main ingredient of Di'ao Xinxuekang Capsules, which is used to prevent and treat coronary heart disease, angina pectoris, and blood stasis caused by palpitations, dizziness, and other syndromes, and is available in the form of capsules. Studies have shown that it can improve myocardial ischemia, reduce myocardial infarction area, improve heart function, and enhance vasoconstriction. Additionally, it can lower blood lipid levels and reduce atherosclerosis, platelet aggregation, and thrombosis. Steroidal saponins, the principal component of Huang yam, have superior antitumor effects and are effective in leukemia and most solid tumors [8]. A meta-analysis showed that Huang yam either alone or in combination with conventional Western medicine treatment and the efficacy of angina have a good effect [9]. In terms of safety, Huang yam alone has a better advantage.

At present, there has been little research on the use of the main components of Huang yam in the treatment of tumors or antitumor drug-induced cardiotoxicity. Therefore, our current study used network pharmacology, molecular docking, and other methods to analyze the potential targets of Huang yam and explore its mechanism of action in tumors and antitumor drug-induced cardiotoxicity.

**Figure 1:**
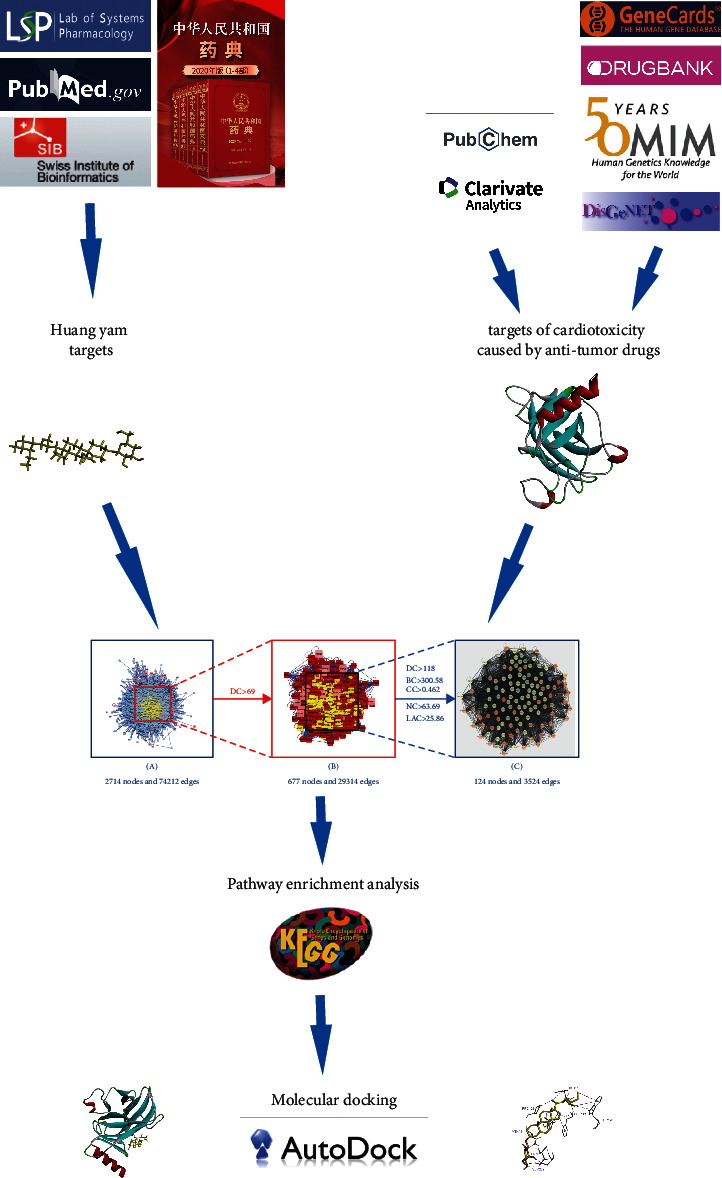
Study framework based on network pharmacology.

Huang yam contains multiple components. Each component has many targets, and there may be synergistic or antagonistic effects among the targets. Therefore, it is difficult to identify the mechanism of action of Huang yam in the treatment of cardiotoxicity caused by antitumor drugs. As network pharmacology is based on the information obtained from various databases, it can provide information on the components of this traditional Chinese medicine (TCM) and its overall mechanism of action in treating diseases. The experimental framework of this research is shown in Figure 1.

## 2. Materials and Methods

### 2.1. Database Search to Obtain Information on the Components of Huang Yam

The keyword “Huang yam” was used to comprehensively search multiple databases to obtain the chemical components of Huang yam. Databases searched included Integrated Pharmacology-based Research Platform of TCM [10], TCM Systems Pharmacology Database and Analysis Platform [11], TCM Molecular Mechanism Bioinformatics Analysis Platform [12], and “Chinese Pharmacopoeia” (2020 edition) [13]. CNKI, Wanfang, Weipu, PubMed, and Web of Science were used for the literature search.

### 2.2. Screening of Active Compounds and Target Prediction of Huang Yam

ChemDraw19.0 [7] was used to draw the chemical structures of the compounds obtained in the database search and convert them into Spatial Data Format files, which were then uploaded to the SwissADME platform [14] (http://www.swissadme.ch/) to search for candidate compounds and predict the related parameters of drug-like properties. In the predicted results, if the gastrointestinal absorption of the compound was “high,” it indicated that the compound had good oral bioavailability; it was assumed that two of the five drug properties (Lipinski, Ghose, Veber, Egan, and Muegge) and two or more compounds are “Yes.” A compound that satisfied both conditions was considered to be an active compound. The SDF format file of the selected active ingredients was imported onto the SwissTargetPrediction platform [15] (http://www.swisstargetprediction.ch/), after which the species name was set to “*Homo sapiens*.” All predicted results were saved and the duplicate values were deleted.

### 2.3. Identification of Targets Related to Cardiotoxicity Caused by Antitumor Drugs

Spatial Data Format files of seven types of anthracyclines drugs (doxorubicin, epirubicin, pirarubicin, aparubicin, icarubicin, amrubicin, and daunorubicin) and tyrosinase inhibitors were retrieved using PubChem [16]. The SwissTargetPrediction platform was used to obtain component targets. Information from the literature complemented the results of the SwissTargetPrediction platform. Anti-human epidermal growth factor receptor (HER-2) drugs and targets related to immune checkpoint blocking therapy were retrieved from the SwissTargetPrediction platform after getting the information from the literature. After combining the results, duplicate values were removed to obtain the target group I. Cardiotoxicity caused by chemotherapeutic drugs mainly included heart failure, arrhythmia, and myocardial ischemia [3], and words related to cardiotoxicity, such as “cardiotoxicity,” “heart failure,” “myocardial ischemia,” and “arrhythmia,” were used in the search. The GeneCards [17], OMIM [18], DrugBank [19], and DisGeNET [20] databases were searched using the keywords to obtain related targets, and the results were merged, after which duplicate values were removed to obtain target group II. These two target groups were intersected to obtain the relevant targets of antitumor drug-induced cardiotoxicity.

### 2.4. Protein-Protein Interaction (PPI) Network Construction and Core Target Screening

A PPI network was constructed using Cytoscape's BisoGenet [21, 22] plug-in. The relevant targets and disease targets of the active ingredients of Huang yam were introduced into BisoGenet, and a PPI network was generated for each. The intersection network of the two PPI networks was extracted through the Merge function in Cytoscape, after which CytoNCA [23] was used to analyze each node in the intersection network, known as the attribute value. The median *k*1 of connectivity was calculated, and all nodes with connectivity greater than 2× *k*1, namely, “Hit hubs,” were selected. The attributes of each node in the Hit hubs network were determined to obtain the degree centrality, closeness centrality, betweenness centrality, network centrality, and local average connectivity. All nodes whose attributes were greater than the five medians, *k*2, *l*2, *m*2, *n*2, and *o*2, were selected as the core targets.

### 2.5. Bioconcentration Analysis

The Metascape platform (http://metascape.org) [24] can be used to perform pathway enrichment analysis on target points. The platform is powerful and integrates multiple authoritative functional databases, such as GO, KEGG, and UniProt, and supports batch gene or protein annotation, enrichment analysis, and PPI network construction. The platform is updated once a month to ensure the reliability of the data. Potential targets were imported into the Metascape platform for GO and KEGG analysis, and the results were saved and visualized using R4.0 software.

### 2.6. Molecular Docking

ChemDraw19.0 [13] was used to draw the structural formulae of the compounds in Huang yam, which were converted into a Spatial Data Format file. The core genes were obtained from the UniProt database [25], and AutoDock Tools 1.5.6 was used for dehydration and hydrogenation. AutoDock Vina was used for docking, after which Discovery Studio 2016 was used to visualize the protein and molecule binding regions.

### 2.7. Identification of the Active Ingredients and Targets of Huang Yam

Thirty-eight compounds of Huang yam were obtained through a literature search using the following databases: TCMIP, China HowNet, and PubMed. Using ChemDraw19.0, the structural formulae of 38 compounds of Huang yam were drawn and converted into SDF files, after retrieving them from the SwissADME website. The targets were obtained using the SwissTargetPrediction platform. The Chinese Pharmacopoeia was searched, after which the active ingredient pseudoprotodioscin was identified as an 11^th^ potential candidate. The relevant target prediction technology was used to predict the target of all 11 identified active ingredients. Duplicate targets were excluded, after which 124 predicted targets were obtained.

### 2.8. Construction of “Active Ingredient-Target” Network

Using Cytoscape 3.7.1, the relationship network between active components and targets of *Dioscorea panthaica* Prain et Burk was drawn and analyzed.

## 3. Results

### 3.1. Identification of Active Ingredients and Targets of Huang Yam

Eleven candidate active compounds were obtained from the SwissTargetPrediction platform described in Section 2.7, as shown in Table 1.

### 3.2. Analysis of the “Active Ingredient-Target” Network

**Figure 2:**
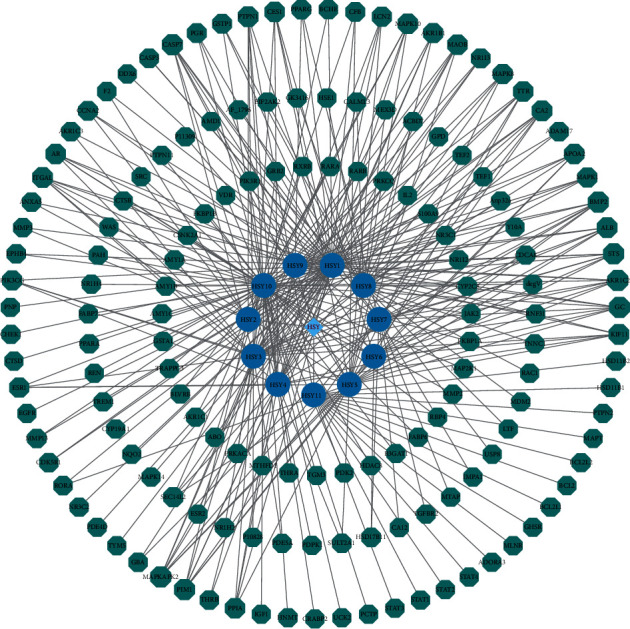
Active ingredients of *Dioscorea panthaica* Prain et Burk. (Note: in the figure, green represents the target of active components; blue represents 11 active components).

The total number of nodes is 139. The node size in Figure 2 represents the size of the degree value. The larger the node area, the greater the degree value. This indicates that the more biological functions involved, the higher the biological importance (Figure 2).

### 3.3. Identification of Targets Related to Cardiotoxicity Caused by Antitumor Drugs

After searching the GeneCards, OMIM, DrugBank, and DisGeNET databases, 2059 targets related to cardiotoxicity caused by antitumor drugs were obtained.

### 3.4. Construction of PPI Network and Screening of Key Targets for Huang Yam to Treat Cardiotoxicity Caused by Antitumor Drugs

#### 3.4.1. PPI Network Drawing of Huang Yam in Treating Cardiotoxicity Caused by Antitumor Drugs

**Figure 3:**
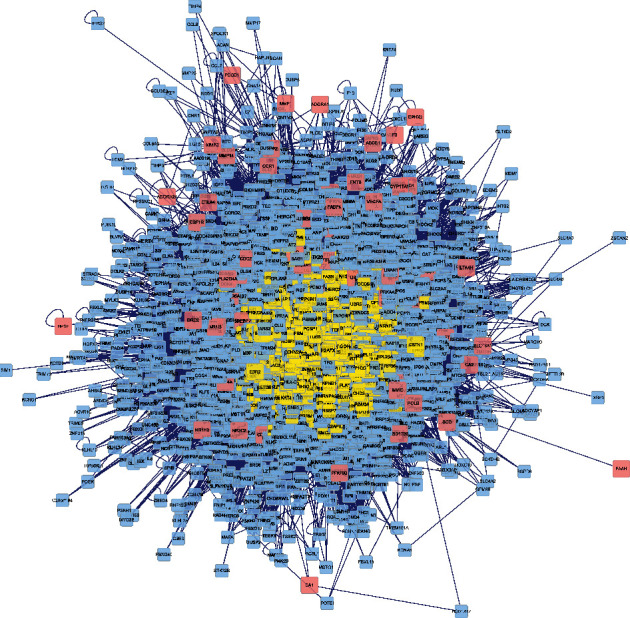
Intersection diagram of the active components of Huang yam and antitumor drug-induced cardiotoxicity-related targets.

It was discovered that the potential targets of Huang yam can interact directly or indirectly with 4557 targets, and the mutual relationships among these targets reached 107,763. PPI network mapping of the targets related to cardiotoxicity caused by antitumor drugs showed that there were 4590 related targets and 114605 interrelationships among these targets. The intersection network of the two is shown in Figure 3.

#### 3.4.2. Key Target Screening

**Figure 4:**
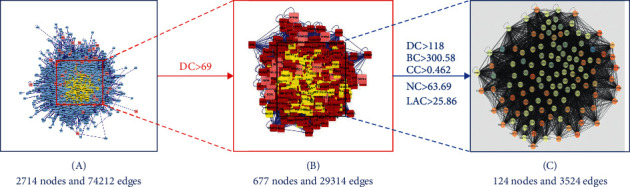
Screening strategy diagram of key nodes. Note: degree centrality (DC), closeness centrality (CC), betweenness centrality (BC), network centrality (NC), and local average connectivity (LAC).

**Figure 5:**
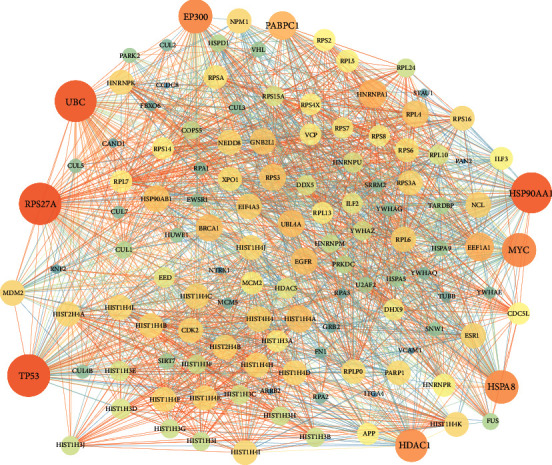
Huang yam core target interaction network.

To obtain richer node connection information in the PPI network, the efficiency of node information transmission was optimized to fully clarify the targets that play an important role in the network. The network topology characteristic attribute values of the abovementioned intersection PPI network graph were calculated. After two screenings, 124 key targets were obtained. The specific screening strategy is shown in Figure 4, and the interaction diagram of the final screening results is shown in Figure 5.

**Figure 6:**
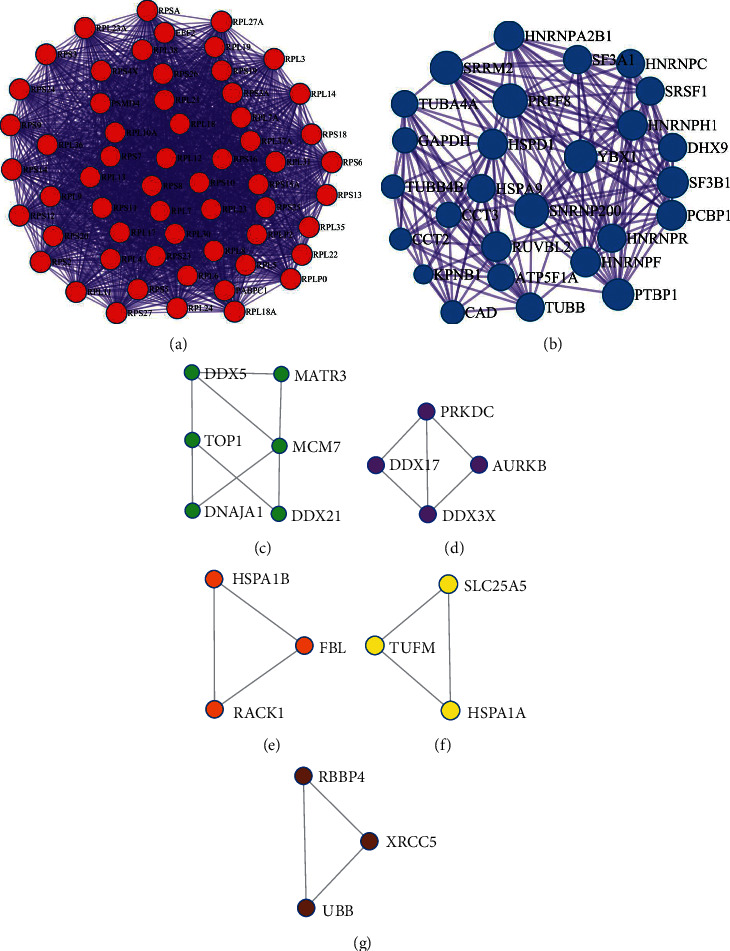
Potential antitumor-induced cardiotoxicity treatment module network within the core PPI network of Huang yam.

As the roles of proteins in the PPI network are mutual, they are usually classified as undirected graphs. The internal network of the module is a potential subnet of the PPI network. The density of the subnet connection was high, whereas that of the regional part of the connection was low. Therefore, the module is considered to have biological significance. This connection has two meanings: first, a complex comprising multiple proteins that play a biological role; secondly, a functional module, such as proteins located in the same pathway, which interact more closely. Therefore, to accurately analyze the mechanism of action of Huang yam in treating cardiotoxicity caused by antitumor drugs, it is necessary to further identify the internal modules of the core PPI network. The interactive relationship was analyzed using a complex molecular detection algorithm, and the module was obtained, as shown in Figure 6. As shown in Figure 6, in the PPI network, the internal connection density of subnetwork 1 is higher, but the ribosomal genes are dominated genes in subnetwork 1. It shows that in this PPI network the ribosomal genes interact more closely with each other than the degree of interaction between ribosomal genes with nonribosomal genes. Subnetwork 2 reflects more biological information. For example, YBX1, which is at the core of the network, is closely related to genes such as SNRNP200 and PRPF8. It is suggested that these genes may have closer cooperation in the treatment of antitumor drug-induced cardiotoxicity by Huang yam. According to the *P* value, the biological processes of the three best scores in the PPI network and the module were retained to describe their functions, as shown in Table 2.

### 3.5. Pathway Enrichment Analysis of Huang Yam in Treating Cardiotoxicity Caused by Antitumor Drugs

**Figure 7:**
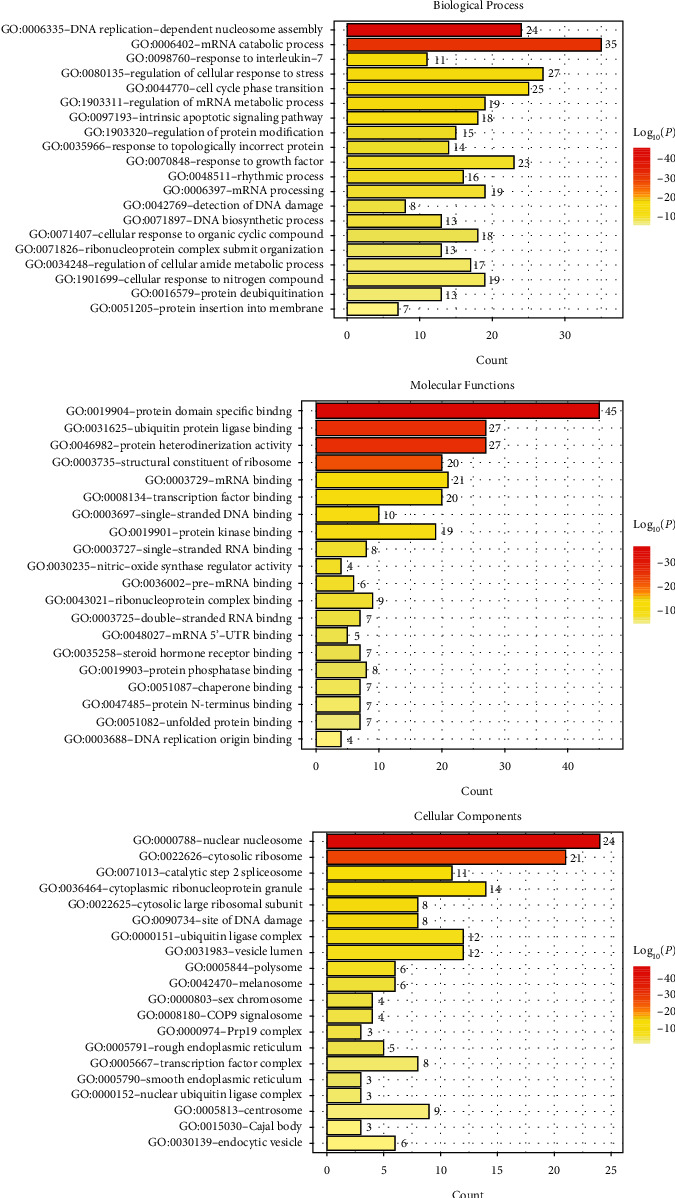
GO analysis diagram of candidate genes of Huang yam for the treatment of antitumor drug-induced cardiotoxicity.

The Metascape platform was used to perform gene enrichment analysis on the above 124 key nodes, including biological process, cellular component, and molecular function in GO and KEGG pathways. The results were saved, after which the R language was used to draw bubbles.  Figure 7 shows the result of the bubble chart.

The GO analysis results of the candidate targets showed that 478 GO terms were enriched, and these were 316 biological processes, 78 cell components, and 84 molecular functions. According to the *P* value after correction before selection, 20 bars were displayed. The left side of the figure shows the names of the top GOs. In biological processes, items such as SRP-dependent cotranslational proteins targeting the cell membrane, ribonucleoprotein complex biogenesis, RNA splicing, and ribonucleoprotein complex assembly were at the forefront. In molecular functions, items such as structural constituents of ribosomes, mRNA binding, protein domain-specific binding, and cadherin binding were at the top. In cellular components, items such as cytosolic ribosomes, cytosolic small ribosomal subunits, polysomal ribosomes, and catalytic step 2 spliceosome were in the dominant position.

**Figure 8:**
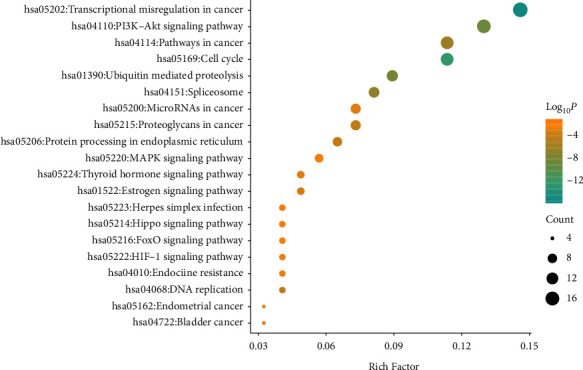
KEGG enrichment analysis diagram of candidate genes for cardiotoxicity induced by anticancer drugs by Huang yam. Note: the vertical axis represents the names of the 7 selected pathways, the color of the dot represents the log_10_(*P*) value, the size represents the number of genes, and the horizontal axis is the ratio of genes.

In KEGG enrichment analysis of candidate targets, 140 pathways were significantly enriched, which are represented using bubble mapping, as shown in Figure 8. These included transcriptional miscegenation in cancer, PI3K-Akt signaling pathway, pathways in cancer, cell cycle, ubiquitin-mediated proteolysis, and other signal transduction pathways.

### 3.6. Molecular Docking Results

**Figure 9:**
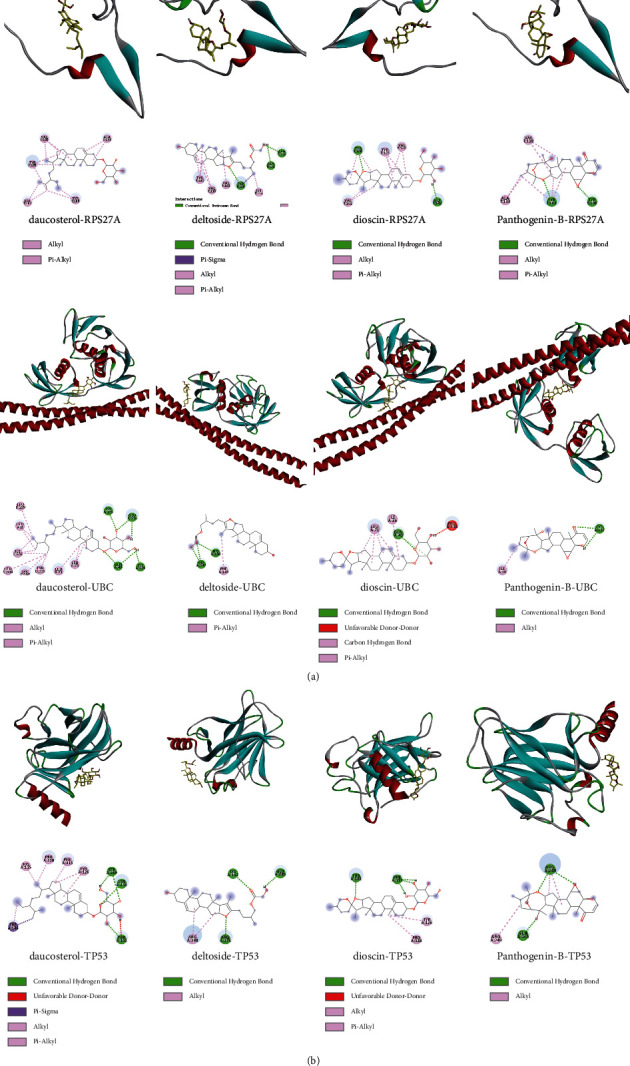
The binding mode diagram of daucosterol, deltoside, dioscin, panthogenin-B, RPS27A, UBC, and TP53.

The top 4 pharmacodynamic molecules with degree value and core genes in the PPI network for molecular docking were selected using AutoDock Vina software. The docking results show (Table 3) that the lowest binding potential energy of the molecule and the target protein was less than 0, indicating that both the ligand and the receptor could bind spontaneously. Among them, the binding energy ranks among the top three distribution positions were as follows: the binding energy of dioscin and TP53 (−41.76 kj/mol), the binding energy of dioscin and UBC (−41.28 kj/mol), and the binding energy of Panthogenin-B and UBC (−39.36 kj/mol) mol). The above docking results were visualized using Discovery Studio 2016 (Figure 9). Daucosterol and TP53 formed hydrogen bonds with SERA269, ASNA131, and THRA102; deltoside and RPS27A formed hydrogen bonds with ARGf116, ALAf128, and TYRf106; and dioscin and UBC formed hydrogen bonds with GLNA40.

## 4. Discussion

TCM is an ancient system of medicine that originated in China and has a history spanning thousands of years. Several studies have shown that the combination of TCM and modern medicine can attenuate the toxicity of tumors. Syndrome differentiation and treatment is one of the core benefits of TCM. Huang yam has the functions of regulating qi (“vital energy”), detoxifying, and relieving pain and swelling. A metastudy showed that Di'aoxinxuekang capsules, the main ingredient of which is Huang yam, are significantly better than isosorbide dinitrate for angina pectoris and can significantly improve symptoms in patients as observed in the ECG [26, 27]. A recent study showed that Huang yam can reduce the blood lipids of ApoE/mice by interfering with the expression of PCSK9 [28]. Other studies have also shown that Huang yam may be useful in treating coronary heart disease by inhibiting lipid peroxidation in the body, thereby improving vascular endothelial function [29]. Even though Huang yam is widely used clinically to treat coronary heart disease and angina pectoris, there are few reports on its use in the treatment of tumors and antitumor drug-induced cardiotoxicity.

### 4.1. Potentially Active Ingredients

From the compound-target network of Huang yam, the top 4 compounds with degree values, namely, daucosterol, deltoside, dioscin, and panthogenin-B, were obtained, with degree values of 60, 58, 31, and 26, respectively. The degree values of daucosterol and deltoside far exceeded those of other ingredients; therefore, these were the potential core compounds with the highest degree of biological activity against tumors and cardiovascular diseases.

Daucosterol can inhibit the migration and invasion of liver cancer cells through the Wnt/*β*-catenin signaling pathway [30]. Additionally, it can induce autophagy in a ROS-dependent manner to inhibit human breast cancer (MCF-7) and gastric cancer (MGC803, BGC823, and AGS) cell proliferation [31]. Furthermore, daucosterol has therapeutic effects on lung cancer cells and no toxic side effects on normal cells [32]. There are not many reports on the effect of daucosterol on cardiotoxicity. More research on it is needed.

Dioscin is a steroidal saponin. Pharmacological experiments have shown that dioscin has antitumor, anti-inflammatory, immune-regulatory, lipid-lowering, antiviral, and antiallergic properties. Furthermore, it has beneficial effects on the heart, liver, kidney, brain, and gastrointestinal system and has broad application prospects [33]. Additionally, dioscin protects against cardiotoxicity caused by doxorubicin by regulating the myocardial oxidative stress response mediated by microRNA-140-5p [34]. Moreover, dioscin may prevent myocardial injury in diabetic rats by upregulating the NO-sGC-cGMP-PKG pathway [35]. It may also protect against coronary heart disease by regulating oxidative stress and inflammation via Sirt1/Nrf2 and p38 MAPK pathways [36] and AngII-induced cardiac hypertrophy via the inhibition of the MAPK and Akt/GSK3*β*/mTOR pathways [37].

Dioscin can protect the liver and nephrotoxicity caused by the anticancer drug, doxorubicin [38]. It inhibits MDR1 expression by downregulating NF-κB signaling in MCF-7 and MCF-7/ADR cells and improves sensitivity to adriamycin. The inhibition of the PI3K/AKT pathway reduces the cytotoxicity of adriamycin [39]. In addition, panthogenin-B is a desstanol steroid, the pharmacological profile of which shows that it is effective against liver cancer and cholangiocarcinoma, and has no toxic side effects on normal cells [40].

### 4.2. GO Biological Process

The GO biological process of core genes is mainly concentrated in the process of protein targeting membrane, RNA shearing, and the assembly and generation of ribonucleoprotein complexes. Huang yam may induce RNA cleavage, interfere with the assembly and generation of ribonucleoprotein complexes, activate Nrf2 and Sirt2 signaling pathways, and reduce HO-1 levels. The expression of NQO1, Gst, GCLM, Keap1, and FOXO3a can interfere with the cardiotoxicity caused by antitumor drugs. Huang yam may intervene in the synthesis of corresponding proteins by influencing the production of ribosomes, thereby intervening in cardiotoxicity caused by antitumor drugs. There are many transcription factors in the gene targets of cardiotoxicity caused by antitumor components in Huang yam. It also involves the binding process of enzymes, transcription factors, and receptors, and achieves therapeutic effects by interfering with the synthesis of these proteins.

### 4.3. Enrichment Analysis of KEGG Signaling Pathway

KEGG enrichment analysis showed that the main regulatory pathways by which Huang yam interfered with anticancer drug-induced cardiotoxicity were abnormal transcription, PI3K-Akt signaling pathway, cell cycle, cancer pathway, and ubiquitination-mediated proteolysis. This indicates that Huang yam not only interferes with the cardiotoxicity caused by antitumor drugs but also plays a positive role in the treatment of cancer through the above pathways and can be used as an adjuvant therapy for cancer chemotherapy.

The activation of the abnormal transcription signal pathway in cancer is related to the abnormal expression of genes related to the cell cycle and cell proliferation, leading to abnormal cell division and the inability of normal cell apoptosis to promote cancer cell proliferation. Cancer cells can use the misregulation mechanism of mRNA to produce proteins with abnormal domains [41], thereby complicating the abnormal cellular proliferation.

As one of the classic signaling pathways, the PI3K-Akt signaling pathway plays a key role in the occurrence and development of many diseases. It mainly affects the survival, apoptosis, metastasis, and infiltration of tumor cells. The specificity of Akt activation is one of the key factors that results in tumor proliferation [42]. The PI3K-Akt signaling pathway also plays a key role in heart disease and can regulate the size, survival, apoptosis, angiogenesis, and inflammatory pathological cardiac hypertrophy of cardiomyopathy [43]. Zhang et al. [44] found that daucosterol can protect cells and reduce apoptosis by inhibiting the PI3K-Akt-mTOR signaling pathway. Han [32, 45] found that daucosterol can inhibit tumor growth by affecting the PI3K-Akt-mTOR signaling pathway.

Changes in the cell cycle are closely related to heart diseases. Cardiomyocyte damage in many heart diseases is irreversible. Eghbali et al. [46] found that changing the signal pathway that affects the cell cycle has a certain improvement effect on mice with myocardial infarction and can restore the myocardial damage caused by anthracyclines to normal. Sun [47] found that regulating the cell cycle can delay the senescence of endothelial cells.

The ubiquitination-mediated proteolysis pathway degrades intracellular proteins to regulate cell activities and plays a vital role in cell proliferation and growth. Xiao et al. [48] believe that the ubiquitination-mediated proteolytic pathway is one of the key factors influencing dilated cardiomyopathy. A study by Nakayama [49] showed that the ubiquitination-mediated proteolytic pathway regulates cell cycle growth factors of many malignant tumor cells. Moreover, Watanabe et al. [50] found that E3-mediated proteolysis can inhibit the expression of ribosomal protein L23 (RPL23), leading to tumors.

## 5. Conclusions

In this study, the method of network pharmacology and molecular docking was used to initially explore the biological mechanism of Huang yam in the treatment of cardiotoxicity caused by anticancer drugs. It was observed that Huang yam achieves therapeutic effects through a complex network of multiple components, pathways, and targets. This study showed that the main active ingredients of Huang yam are daucosterol, deltoside, dioscin, and Panthogenin-B, which mainly target TP53, RPS27A, and UBC. The main regulatory pathways were abnormal transcription, PI3K-Akt signaling pathway, cell cycle, cancer pathway, and ubiquitination-mediated proteolysis.

This study showed that Huang yam may be effective in treating heart diseases caused by antitumor therapy by inhibiting ubiquitination-mediated proteolysis, PI3K-Akt signaling pathway, and cancer pathway. According to the research and prediction of network pharmacology, we found that Huang yam is not only efficacious in reducing cardiotoxicity caused by anticancer drugs but also has a positive effect on the treatment of tumors. Therefore, it may be a potential drug for tumor treatment.

The results of this study have been generated using a theoretical approach and the real-life situation may be slightly different. Thus, further studies using *in vitro* and *in vivo* experiments are required to corroborate our findings.

## Figures and Tables

**Table 1:** Candidate active components in *Dioscorea panthaica* Prain et Burk.

ID	Compound	CAS	Chemical formula	Structure type
HSY1	Diosgenin	512-04-9	C_27_H_42_O_3_	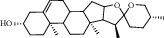
HSY2	Pseudoprotodioscin	102115-79-7	C_51_H_82_O_21_	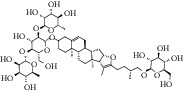
HSY3	3*β*,26-Diol-25 (R)-Δ5, 20 (22)-diene-furosta-26-O-*β*-D-glucopyranoside	—	C_29_H_44_O_5_	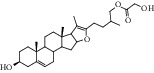
HSY4	1, 7-Bis (4-hydroxyphenyl) hepta-4E, 6E-dien-3-one	332371-82-1	C_19_H_18_O_3_	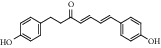
HSY5	Progenin III (3-O-[*α*-L-rhamnopyranosyl (1 ⟶ 2)]-*β*-D-glucopyranoside-diosgenin)	19057-67-1	C_39_H_62_O_12_	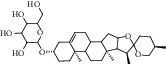
HSY6	Trillin	14144-06-0	C_33_H_52_O_8_	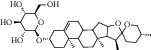
HSY7	Beta-sitosterol	64997-52-0	C_30_H_52_O	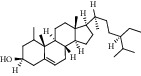
HSY8	Daucosterol	474-58-8	C_35_H_60_O_6_	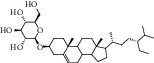
HSY9	Palmiticacid	57-10-3	C_16_H_32_O_2_	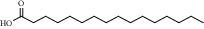
HSY10	Panthogenin-A	—	C_27_H_40_O_7_	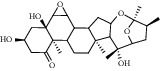
HSY11	Panthogenin-B	—	C_27_H_38_O_6_	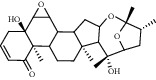

**Table 2:** Functional description of potential modules within the core PPI network of Huang yam for the treatment of cardiotoxicity caused by antitumor drugs (top 3).

Term	Description	Log_10_(*P*)
hsa03010	Ribosome	−88.71047413
hsa03010	Ribosome	−88.71047413
hsa05203	Viral carcinogenesis	−14.60269766

**Table 3:** Binding energy results of core components in Huang yam with TP53, RPS27A, and UBC.

Compound	Chemical formula	Relative molecular mass (g/mol)	TP53 (kJ/mol)	RPS27A (kJ/mol)	UBC (kJ/mol)
Daucosterol	C_35_H_60_O_6_	576.85	−36.96	−33.81	−36
Deltoside	C_29_H_44_O_5_	472.66	−31.2	−33.6	−33.6
Dioscin	C_33_H_52_O_8_	576.76	−41.76	−37.44	−41.28
Panthogenin-B	C_27_H_38_O_6_	458.59	−37.92	−35.52	−39.36

## Data Availability

This study's data and materials will be made available by the corresponding author upon a reasonable request.

## Supplementary Materials

Supplementary Materials 1. Three-dimensional structure of diosgenin/HSY1, which is a candidate active compound of Huang yam. 2. Three-dimensional structure of pseudoprotodioscin/HSY2, which is a candidate active compound of Huang yam. 3. Three-dimensional structure of 3*β*, 26-diol-25 (R)-∆5, 20 (22)-diene-furosta-26-O-*β*-D-glucopyranoside/HSY3, which is a candidate active compound of Huang yam. 4. Three-dimensional structure of 1, 7-bis (4-hydroxyphenyl) hepta-4E, 6E-dien-3-one/HSY4, which is a candidate active compound of Huang yam. 5. Three-dimensional structure of progenin (3-O-*α*-L-rhamnopyranosyl (1 ⟶ 2)]-*β*-D-glucopyranoside-diosgenin)/HSY5, which is a candidate active compound of Huang yam. 6. Three-dimensional structure of trillin/HSY6, which is a candidate active compound of Huang yam. 7. Three-dimensional structure of beta-sitosterol/HSY7, which is a candidate active compound of Huang yam. 8. Three-dimensional structure of daucosterol/HSY8, which is a candidate active compound of Huang yam. 9. Three-dimensional structure of palmitic acid/HSY9, which is a candidate active compound of Huang yam. 10. Three-dimensional structure of panthogenin-A/HSY10, which is a candidate active compound of Huang yam. 11. Three-dimensional structure of panthogenin-B/HSY11, which is a candidate active compound of Huang yam.
