# Supplementary figures and images for: Exploring the Biological Mechanism of Huang Yam in Treating Tumors and Preventing Antitumor Drug-Induced Cardiotoxicity Using Network Pharmacology and Molecular Docking Technology
Source: Evid Based Complement Alternat Med. 2021 Aug 25;2021:9988650. doi: 10.1155/2021/9988650 (PMC8410425; doi:10.1155/2021/9988650)

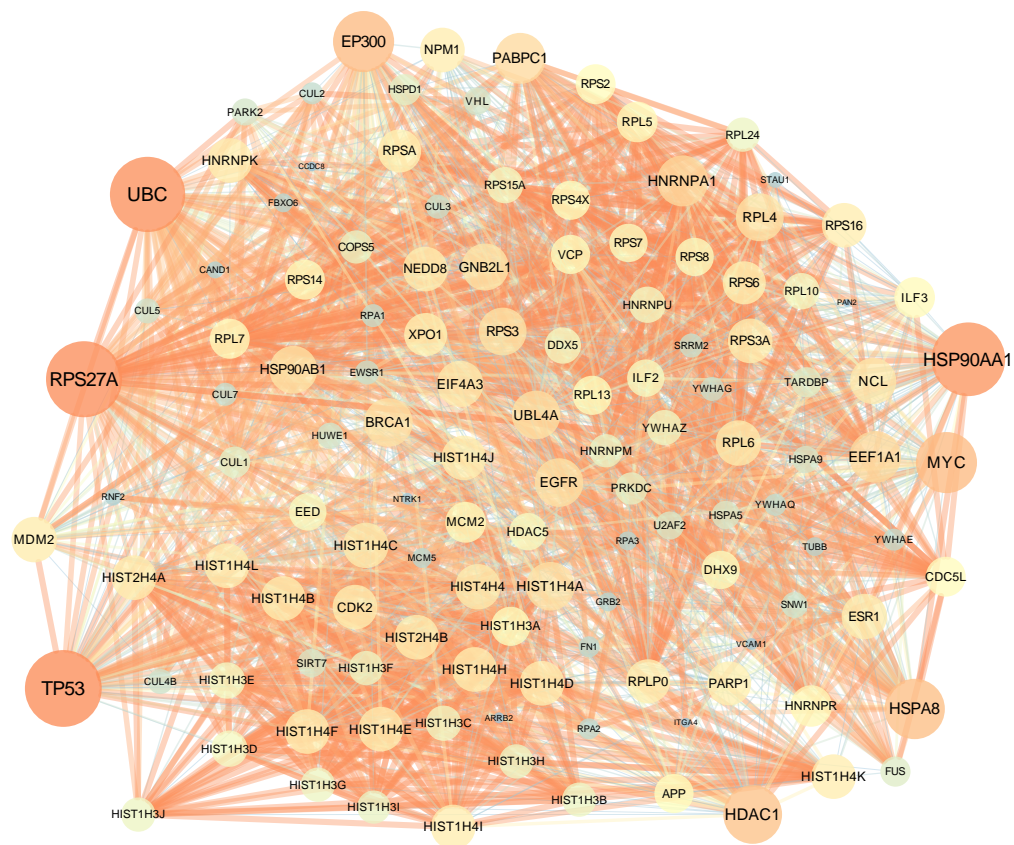

Supplement: Supplementary Materials — 1. Three-dimensional structure of diosgenin/HSY1, which is a candidate active compound of Huang yam. 2. Three-dimensional structure of pseudoprotodioscin/HSY2, which is a candidate active compound of Huang yam. 3. Three-dimensional structure of 3β, 26-diol-25 (R)-∆5, 20 (22)-diene-furosta-26-O-β-D-glucopyranoside/HSY3, which is a candidate active compound of Huang yam. 4. Three-dimensional structure of 1, 7-bis (4-hydroxyphenyl) hepta-4E, 6E-dien-3-one/HSY4, which is a candidate active compound of Huang yam. 5. Three-dimensional structure of progenin (3-O-α-L-rhamnopyranosyl (1 ⟶ 2)]-β-D-glucopyranoside-diosgenin)/HSY5, which is a candidate active compound of Huang yam. 6. Three-dimensional structure of trillin/HSY6, which is a candidate active compound of Huang yam. 7. Three-dimensional structure of beta-sitosterol/HSY7, which is a candidate active compound of Huang yam. 8. Three-dimensional structure of daucosterol/HSY8, which is a candidate active compound of Huang yam. 9. Three-dimensional structure of palmitic acid/HSY9, which is a candidate active compound of Huang yam. 10. Three-dimensional structure of panthogenin-A/HSY10, which is a candidate active compound of Huang yam. 11. Three-dimensional structure of panthogenin-B/HSY11, which is a candidate active compound of Huang yam. [file 9988650.f1.zip › 9988650.f1/Figure 5--Huang yam core target interaction network.pdf]

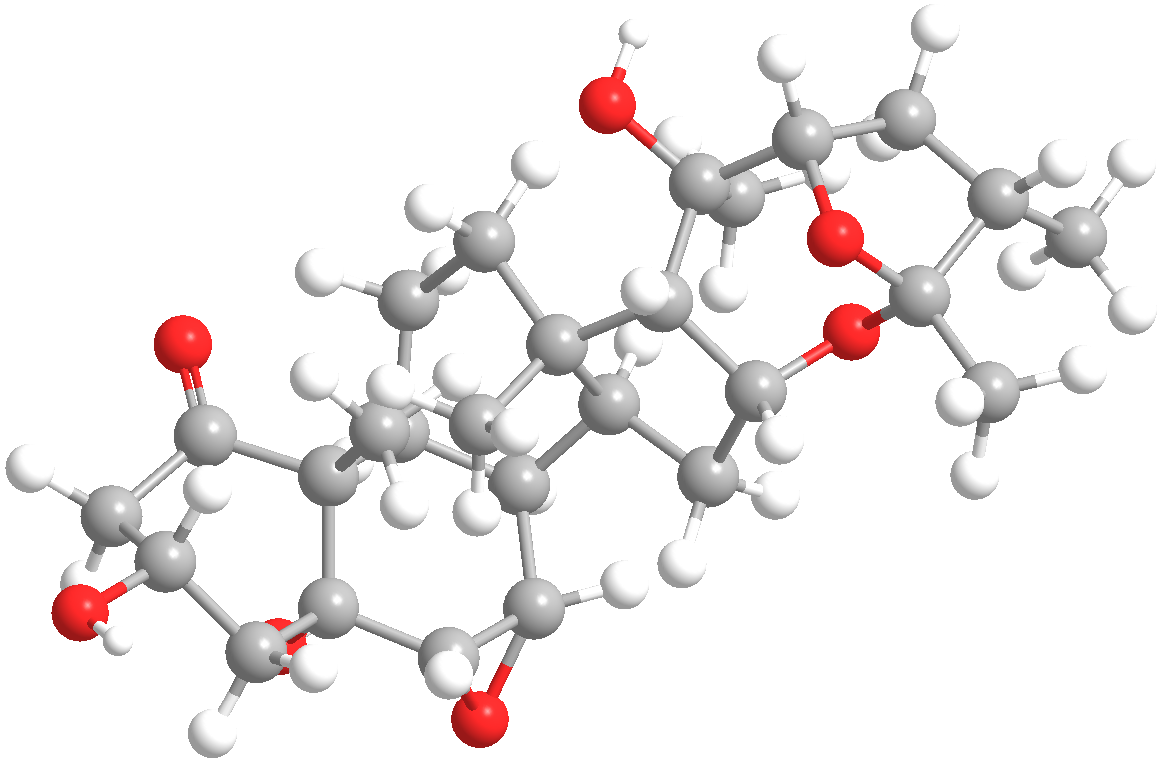

Supplement: Supplementary Materials — 1. Three-dimensional structure of diosgenin/HSY1, which is a candidate active compound of Huang yam. 2. Three-dimensional structure of pseudoprotodioscin/HSY2, which is a candidate active compound of Huang yam. 3. Three-dimensional structure of 3β, 26-diol-25 (R)-∆5, 20 (22)-diene-furosta-26-O-β-D-glucopyranoside/HSY3, which is a candidate active compound of Huang yam. 4. Three-dimensional structure of 1, 7-bis (4-hydroxyphenyl) hepta-4E, 6E-dien-3-one/HSY4, which is a candidate active compound of Huang yam. 5. Three-dimensional structure of progenin (3-O-α-L-rhamnopyranosyl (1 ⟶ 2)]-β-D-glucopyranoside-diosgenin)/HSY5, which is a candidate active compound of Huang yam. 6. Three-dimensional structure of trillin/HSY6, which is a candidate active compound of Huang yam. 7. Three-dimensional structure of beta-sitosterol/HSY7, which is a candidate active compound of Huang yam. 8. Three-dimensional structure of daucosterol/HSY8, which is a candidate active compound of Huang yam. 9. Three-dimensional structure of palmitic acid/HSY9, which is a candidate active compound of Huang yam. 10. Three-dimensional structure of panthogenin-A/HSY10, which is a candidate active compound of Huang yam. 11. Three-dimensional structure of panthogenin-B/HSY11, which is a candidate active compound of Huang yam. [file 9988650.f1.zip › 9988650.f1/HSY10-panthogenin-A.png]

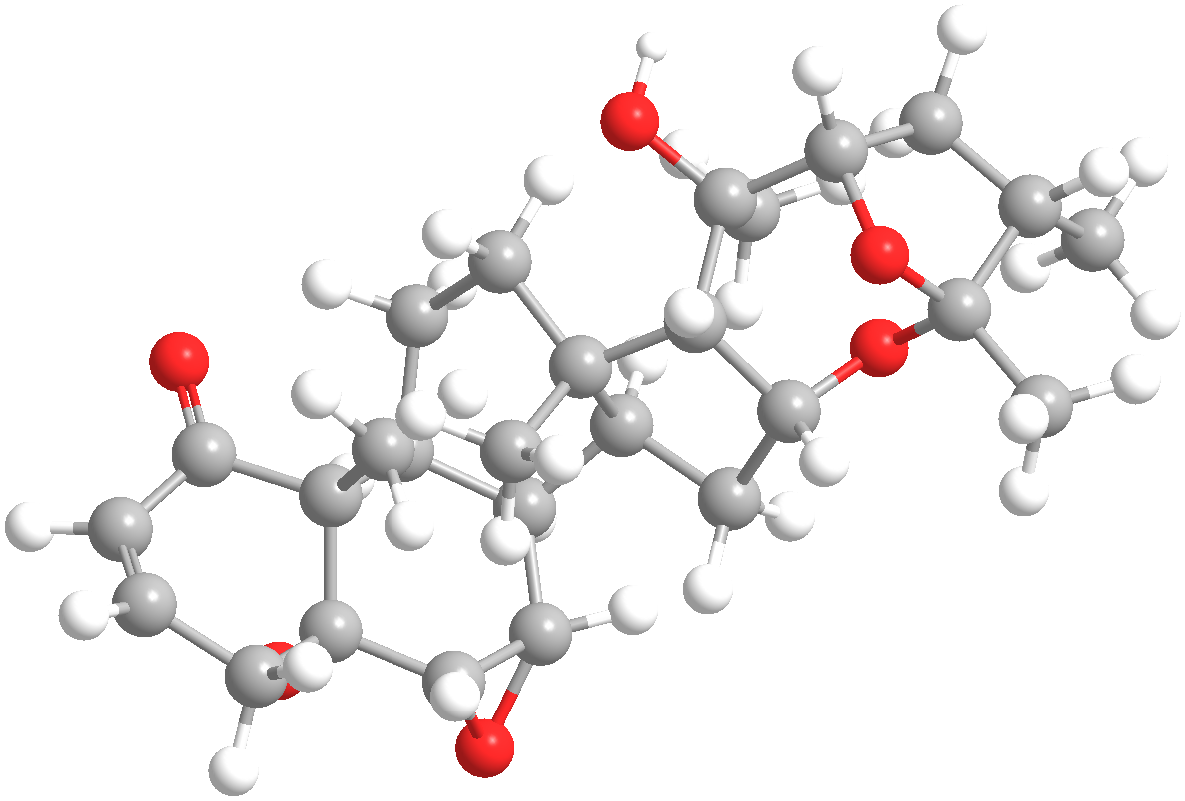

Supplement: Supplementary Materials — 1. Three-dimensional structure of diosgenin/HSY1, which is a candidate active compound of Huang yam. 2. Three-dimensional structure of pseudoprotodioscin/HSY2, which is a candidate active compound of Huang yam. 3. Three-dimensional structure of 3β, 26-diol-25 (R)-∆5, 20 (22)-diene-furosta-26-O-β-D-glucopyranoside/HSY3, which is a candidate active compound of Huang yam. 4. Three-dimensional structure of 1, 7-bis (4-hydroxyphenyl) hepta-4E, 6E-dien-3-one/HSY4, which is a candidate active compound of Huang yam. 5. Three-dimensional structure of progenin (3-O-α-L-rhamnopyranosyl (1 ⟶ 2)]-β-D-glucopyranoside-diosgenin)/HSY5, which is a candidate active compound of Huang yam. 6. Three-dimensional structure of trillin/HSY6, which is a candidate active compound of Huang yam. 7. Three-dimensional structure of beta-sitosterol/HSY7, which is a candidate active compound of Huang yam. 8. Three-dimensional structure of daucosterol/HSY8, which is a candidate active compound of Huang yam. 9. Three-dimensional structure of palmitic acid/HSY9, which is a candidate active compound of Huang yam. 10. Three-dimensional structure of panthogenin-A/HSY10, which is a candidate active compound of Huang yam. 11. Three-dimensional structure of panthogenin-B/HSY11, which is a candidate active compound of Huang yam. [file 9988650.f1.zip › 9988650.f1/HSY11-panthogenin-B.png]

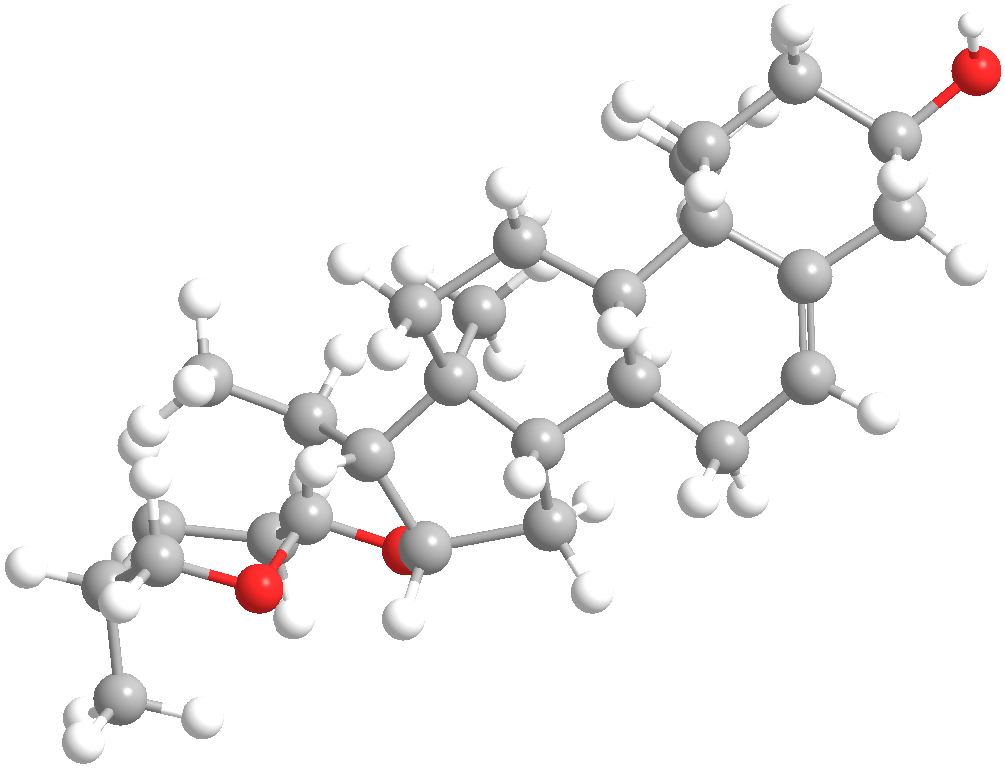

Supplement: Supplementary Materials — 1. Three-dimensional structure of diosgenin/HSY1, which is a candidate active compound of Huang yam. 2. Three-dimensional structure of pseudoprotodioscin/HSY2, which is a candidate active compound of Huang yam. 3. Three-dimensional structure of 3β, 26-diol-25 (R)-∆5, 20 (22)-diene-furosta-26-O-β-D-glucopyranoside/HSY3, which is a candidate active compound of Huang yam. 4. Three-dimensional structure of 1, 7-bis (4-hydroxyphenyl) hepta-4E, 6E-dien-3-one/HSY4, which is a candidate active compound of Huang yam. 5. Three-dimensional structure of progenin (3-O-α-L-rhamnopyranosyl (1 ⟶ 2)]-β-D-glucopyranoside-diosgenin)/HSY5, which is a candidate active compound of Huang yam. 6. Three-dimensional structure of trillin/HSY6, which is a candidate active compound of Huang yam. 7. Three-dimensional structure of beta-sitosterol/HSY7, which is a candidate active compound of Huang yam. 8. Three-dimensional structure of daucosterol/HSY8, which is a candidate active compound of Huang yam. 9. Three-dimensional structure of palmitic acid/HSY9, which is a candidate active compound of Huang yam. 10. Three-dimensional structure of panthogenin-A/HSY10, which is a candidate active compound of Huang yam. 11. Three-dimensional structure of panthogenin-B/HSY11, which is a candidate active compound of Huang yam. [file 9988650.f1.zip › 9988650.f1/HSY1-diosgenin.png]

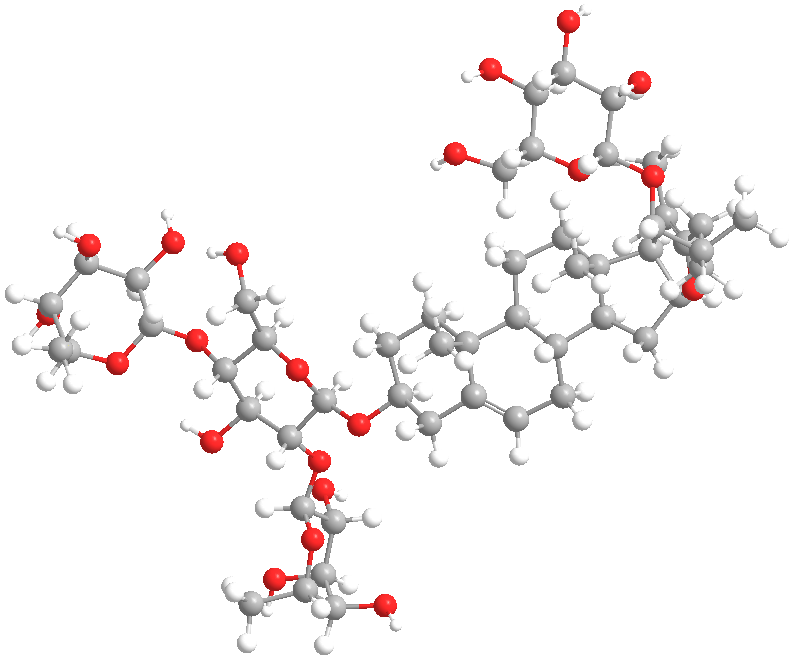

Supplement: Supplementary Materials — 1. Three-dimensional structure of diosgenin/HSY1, which is a candidate active compound of Huang yam. 2. Three-dimensional structure of pseudoprotodioscin/HSY2, which is a candidate active compound of Huang yam. 3. Three-dimensional structure of 3β, 26-diol-25 (R)-∆5, 20 (22)-diene-furosta-26-O-β-D-glucopyranoside/HSY3, which is a candidate active compound of Huang yam. 4. Three-dimensional structure of 1, 7-bis (4-hydroxyphenyl) hepta-4E, 6E-dien-3-one/HSY4, which is a candidate active compound of Huang yam. 5. Three-dimensional structure of progenin (3-O-α-L-rhamnopyranosyl (1 ⟶ 2)]-β-D-glucopyranoside-diosgenin)/HSY5, which is a candidate active compound of Huang yam. 6. Three-dimensional structure of trillin/HSY6, which is a candidate active compound of Huang yam. 7. Three-dimensional structure of beta-sitosterol/HSY7, which is a candidate active compound of Huang yam. 8. Three-dimensional structure of daucosterol/HSY8, which is a candidate active compound of Huang yam. 9. Three-dimensional structure of palmitic acid/HSY9, which is a candidate active compound of Huang yam. 10. Three-dimensional structure of panthogenin-A/HSY10, which is a candidate active compound of Huang yam. 11. Three-dimensional structure of panthogenin-B/HSY11, which is a candidate active compound of Huang yam. [file 9988650.f1.zip › 9988650.f1/HSY2-pseudoprotodioscin.png]

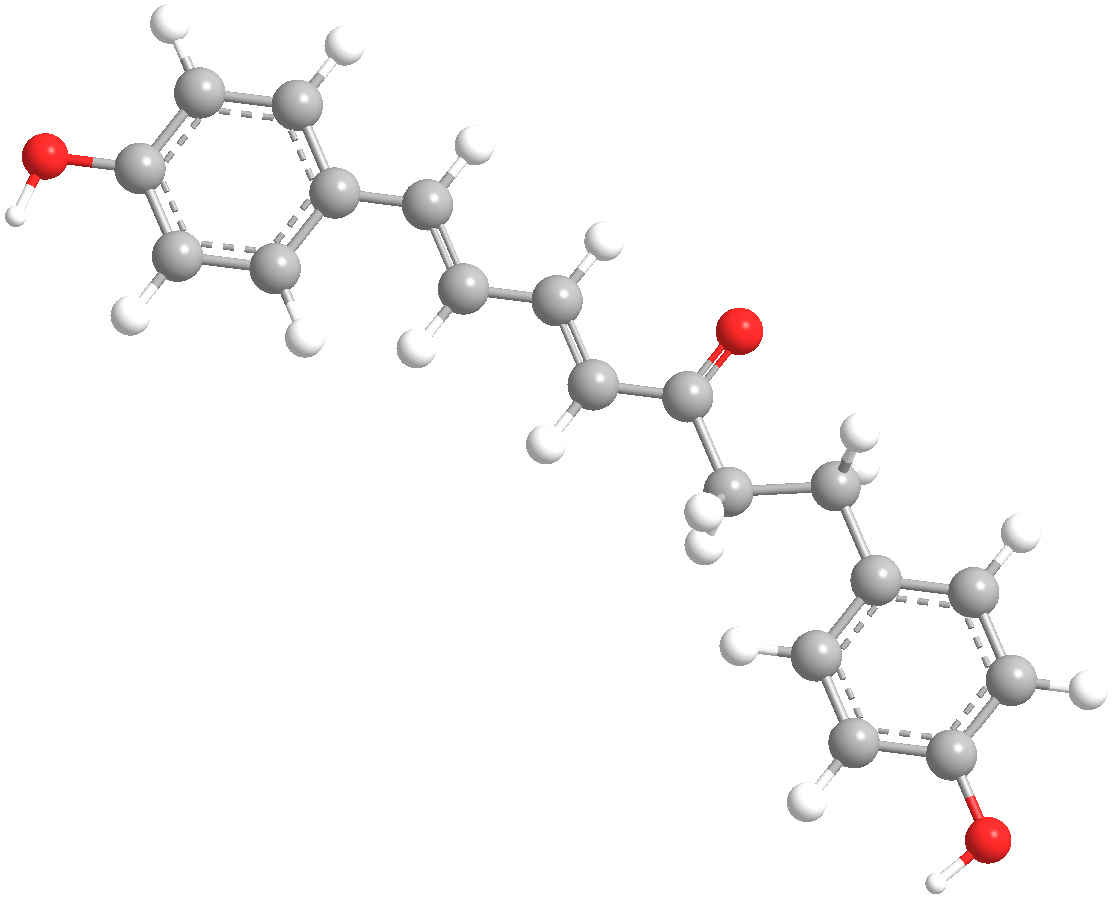

Supplement: Supplementary Materials — 1. Three-dimensional structure of diosgenin/HSY1, which is a candidate active compound of Huang yam. 2. Three-dimensional structure of pseudoprotodioscin/HSY2, which is a candidate active compound of Huang yam. 3. Three-dimensional structure of 3β, 26-diol-25 (R)-∆5, 20 (22)-diene-furosta-26-O-β-D-glucopyranoside/HSY3, which is a candidate active compound of Huang yam. 4. Three-dimensional structure of 1, 7-bis (4-hydroxyphenyl) hepta-4E, 6E-dien-3-one/HSY4, which is a candidate active compound of Huang yam. 5. Three-dimensional structure of progenin (3-O-α-L-rhamnopyranosyl (1 ⟶ 2)]-β-D-glucopyranoside-diosgenin)/HSY5, which is a candidate active compound of Huang yam. 6. Three-dimensional structure of trillin/HSY6, which is a candidate active compound of Huang yam. 7. Three-dimensional structure of beta-sitosterol/HSY7, which is a candidate active compound of Huang yam. 8. Three-dimensional structure of daucosterol/HSY8, which is a candidate active compound of Huang yam. 9. Three-dimensional structure of palmitic acid/HSY9, which is a candidate active compound of Huang yam. 10. Three-dimensional structure of panthogenin-A/HSY10, which is a candidate active compound of Huang yam. 11. Three-dimensional structure of panthogenin-B/HSY11, which is a candidate active compound of Huang yam. [file 9988650.f1.zip › 9988650.f1/HSY4-1,7-bis(4-hydroxyphenyl)hepta-4E,6E-dien-3-one.png]

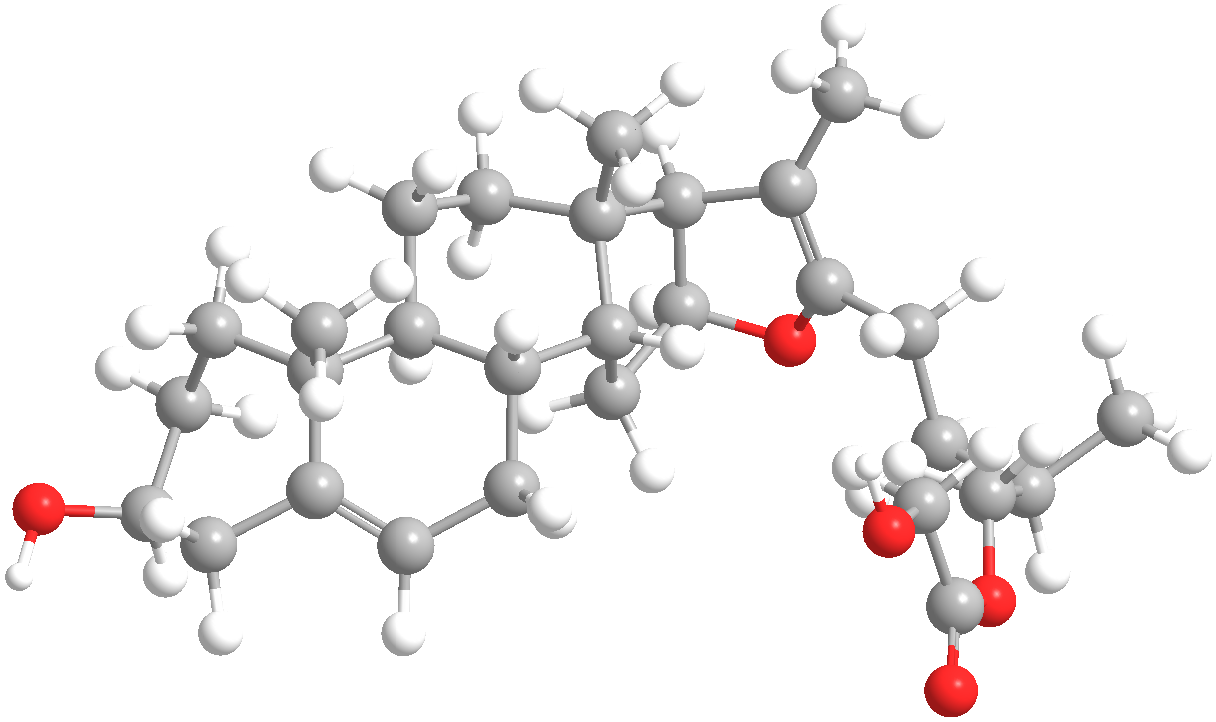

Supplement: Supplementary Materials — 1. Three-dimensional structure of diosgenin/HSY1, which is a candidate active compound of Huang yam. 2. Three-dimensional structure of pseudoprotodioscin/HSY2, which is a candidate active compound of Huang yam. 3. Three-dimensional structure of 3β, 26-diol-25 (R)-∆5, 20 (22)-diene-furosta-26-O-β-D-glucopyranoside/HSY3, which is a candidate active compound of Huang yam. 4. Three-dimensional structure of 1, 7-bis (4-hydroxyphenyl) hepta-4E, 6E-dien-3-one/HSY4, which is a candidate active compound of Huang yam. 5. Three-dimensional structure of progenin (3-O-α-L-rhamnopyranosyl (1 ⟶ 2)]-β-D-glucopyranoside-diosgenin)/HSY5, which is a candidate active compound of Huang yam. 6. Three-dimensional structure of trillin/HSY6, which is a candidate active compound of Huang yam. 7. Three-dimensional structure of beta-sitosterol/HSY7, which is a candidate active compound of Huang yam. 8. Three-dimensional structure of daucosterol/HSY8, which is a candidate active compound of Huang yam. 9. Three-dimensional structure of palmitic acid/HSY9, which is a candidate active compound of Huang yam. 10. Three-dimensional structure of panthogenin-A/HSY10, which is a candidate active compound of Huang yam. 11. Three-dimensional structure of panthogenin-B/HSY11, which is a candidate active compound of Huang yam. [file 9988650.f1.zip › 9988650.f1/HSY6-trillin.png]

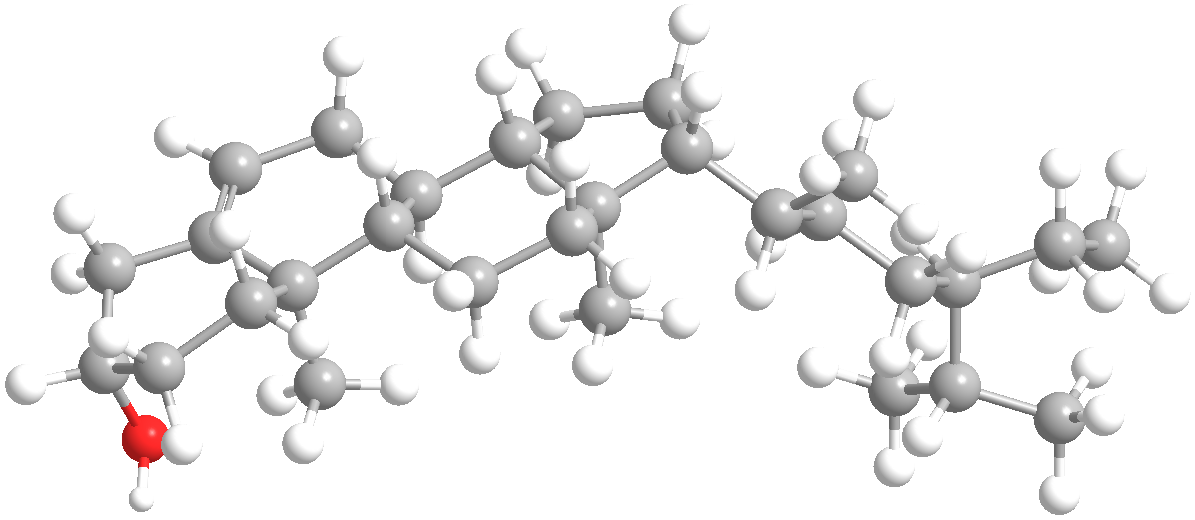

Supplement: Supplementary Materials — 1. Three-dimensional structure of diosgenin/HSY1, which is a candidate active compound of Huang yam. 2. Three-dimensional structure of pseudoprotodioscin/HSY2, which is a candidate active compound of Huang yam. 3. Three-dimensional structure of 3β, 26-diol-25 (R)-∆5, 20 (22)-diene-furosta-26-O-β-D-glucopyranoside/HSY3, which is a candidate active compound of Huang yam. 4. Three-dimensional structure of 1, 7-bis (4-hydroxyphenyl) hepta-4E, 6E-dien-3-one/HSY4, which is a candidate active compound of Huang yam. 5. Three-dimensional structure of progenin (3-O-α-L-rhamnopyranosyl (1 ⟶ 2)]-β-D-glucopyranoside-diosgenin)/HSY5, which is a candidate active compound of Huang yam. 6. Three-dimensional structure of trillin/HSY6, which is a candidate active compound of Huang yam. 7. Three-dimensional structure of beta-sitosterol/HSY7, which is a candidate active compound of Huang yam. 8. Three-dimensional structure of daucosterol/HSY8, which is a candidate active compound of Huang yam. 9. Three-dimensional structure of palmitic acid/HSY9, which is a candidate active compound of Huang yam. 10. Three-dimensional structure of panthogenin-A/HSY10, which is a candidate active compound of Huang yam. 11. Three-dimensional structure of panthogenin-B/HSY11, which is a candidate active compound of Huang yam. [file 9988650.f1.zip › 9988650.f1/HSY7-beta-Sitosterol.png]

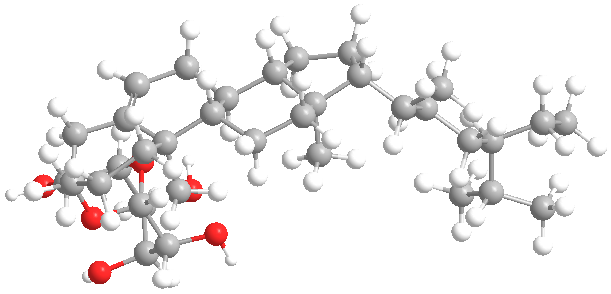

Supplement: Supplementary Materials — 1. Three-dimensional structure of diosgenin/HSY1, which is a candidate active compound of Huang yam. 2. Three-dimensional structure of pseudoprotodioscin/HSY2, which is a candidate active compound of Huang yam. 3. Three-dimensional structure of 3β, 26-diol-25 (R)-∆5, 20 (22)-diene-furosta-26-O-β-D-glucopyranoside/HSY3, which is a candidate active compound of Huang yam. 4. Three-dimensional structure of 1, 7-bis (4-hydroxyphenyl) hepta-4E, 6E-dien-3-one/HSY4, which is a candidate active compound of Huang yam. 5. Three-dimensional structure of progenin (3-O-α-L-rhamnopyranosyl (1 ⟶ 2)]-β-D-glucopyranoside-diosgenin)/HSY5, which is a candidate active compound of Huang yam. 6. Three-dimensional structure of trillin/HSY6, which is a candidate active compound of Huang yam. 7. Three-dimensional structure of beta-sitosterol/HSY7, which is a candidate active compound of Huang yam. 8. Three-dimensional structure of daucosterol/HSY8, which is a candidate active compound of Huang yam. 9. Three-dimensional structure of palmitic acid/HSY9, which is a candidate active compound of Huang yam. 10. Three-dimensional structure of panthogenin-A/HSY10, which is a candidate active compound of Huang yam. 11. Three-dimensional structure of panthogenin-B/HSY11, which is a candidate active compound of Huang yam. [file 9988650.f1.zip › 9988650.f1/HSY8-daucosterol.png]

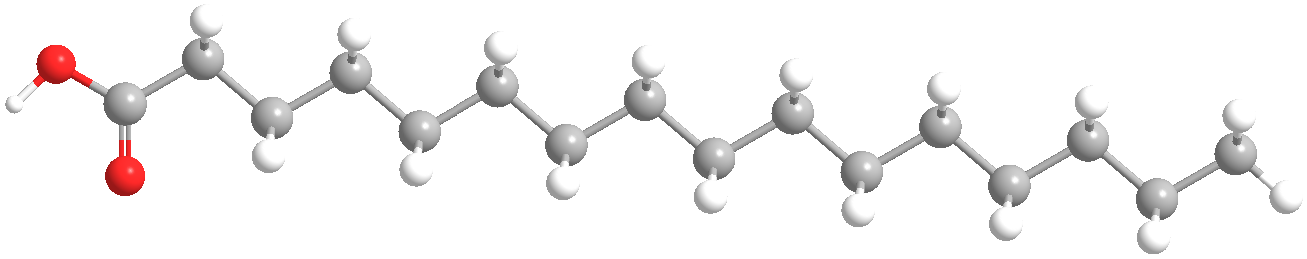

Supplement: Supplementary Materials — 1. Three-dimensional structure of diosgenin/HSY1, which is a candidate active compound of Huang yam. 2. Three-dimensional structure of pseudoprotodioscin/HSY2, which is a candidate active compound of Huang yam. 3. Three-dimensional structure of 3β, 26-diol-25 (R)-∆5, 20 (22)-diene-furosta-26-O-β-D-glucopyranoside/HSY3, which is a candidate active compound of Huang yam. 4. Three-dimensional structure of 1, 7-bis (4-hydroxyphenyl) hepta-4E, 6E-dien-3-one/HSY4, which is a candidate active compound of Huang yam. 5. Three-dimensional structure of progenin (3-O-α-L-rhamnopyranosyl (1 ⟶ 2)]-β-D-glucopyranoside-diosgenin)/HSY5, which is a candidate active compound of Huang yam. 6. Three-dimensional structure of trillin/HSY6, which is a candidate active compound of Huang yam. 7. Three-dimensional structure of beta-sitosterol/HSY7, which is a candidate active compound of Huang yam. 8. Three-dimensional structure of daucosterol/HSY8, which is a candidate active compound of Huang yam. 9. Three-dimensional structure of palmitic acid/HSY9, which is a candidate active compound of Huang yam. 10. Three-dimensional structure of panthogenin-A/HSY10, which is a candidate active compound of Huang yam. 11. Three-dimensional structure of panthogenin-B/HSY11, which is a candidate active compound of Huang yam. [file 9988650.f1.zip › 9988650.f1/HSY9-palmiticacid.png]
